# Supplementary material for: Engineered kinases as a tool for phosphorylation of selected targets in vivo
Source: J Cell Biol. 2022 Sep 14;221(10):e202106179. doi: 10.1083/jcb.202106179 (PMC9477969; doi:10.1083/jcb.202106179)
Supplement: Table S1 — shows the viability and/or adult fly phenotypes induced by the expression of different synthetic kinase constructs in the absence of the GFP-target. [file JCB_202106179_TableS1.docx]

|  | Effector transgene | | | | |
| --- | --- | --- | --- | --- | --- |
| Gal4 driver | ***Rok-cat^T2A^*** | ***N-Rok::vhhGFP4^ZH-86Fb^*** | ***Rok-CAT^3.1^*** | ***N-Rok::vhhGFP4^Vi^*** | ***N-RokDead::vhhGFP4^ZH-86Fb^*** |
| ***tubGal4*** | lethal | lethal | lethal | lethal | wild type |
| ***enGal4*** | lethal | lethal | missing crossveins | partially missing crossveins | wild type |
| ***daGal4*** | lethal | lethal | wild type | wild type | wild type |
| ***69BGal4*** | lethal | semi lethal | missing crossveins | wild type | wild type |
| ***elavGal4*** | rough eyes | rough eyes | wild type | wild type | wild type |

Supplementary Table 1

**Viability and/or adult fly phenotypes induced by the expression of different synthetic kinase constructs with several Gal4 lines in the absence of the GFP-target.**

For every non-lethal condition, more than 20 adult flies were generated with consistent phenotypes. The genomic insertions and the Gal4 drivers were respectively sorted from left to right and top to bottom in descending strength of the phenotypes they induced. See text for details.
