# Supplementary material for: Engineered kinases as a tool for phosphorylation of selected targets in vivo
Source: J Cell Biol. 2022 Sep 14;221(10):e202106179. doi: 10.1083/jcb.202106179 (PMC9477969; doi:10.1083/jcb.202106179)
Supplement: Table S2 — is a summary of protein binders used in this study. [file JCB_202106179_TableS2.docx]

| **Protein binder used** | **description** | **reference** |
| --- | --- | --- |
| vhhGFP4 | *a nanobody directed against GFP and its close derivatives* | (Saerens et al. 2005) |
| 2m22 | *anti-mCherry DARPin* | (Brauchle et al. 2014) |
| dGBP1 | *a destabilized GFP-binding nanobody* | (Tang et al. 2016) |
| 3G86.32 | *anti-GFP DARPin* | (Brauchle et al. 2014) |

Supplementary Table 2

**Summary of protein binders used in this study.**
