# Supplementary material for: Engineered kinases as a tool for phosphorylation of selected targets in vivo
Source: J Cell Biol. 2022 Sep 14;221(10):e202106179. doi: 10.1083/jcb.202106179 (PMC9477969; doi:10.1083/jcb.202106179)
Supplement: Table S3 — contains detailed information on the reagents, genetically modified organisms, and cell lines used. [file JCB_202106179_TableS3.docx]

**KEY RESOURCES TABLE**

| REAGENT or RESOURCE | SOURCE | IDENTIFIER | ADDITIONAL INFO |
| --- | --- | --- | --- |
| **Antibodies** | | | |
| rabbit anti-phospho-Myosin Light Chain 2 (Ser19) | Cell Signaling Technology | #3671; RRID: [AB_330248](http://antibodyregistry.org/AB_330248) | 1:50 |
| mouse anti-HA | Roche | #12CA5, [AB_2532070](http://antibodyregistry.org/AB_2532070) | 1:200 |
| rat anti-Sqh2P | ^1^ |  | 1:200 |
| rabbit anti-Sqh1P | ^1^ |  | 1:50 |
| guinea pig anti-Sqh1P antibody serum in 50% glycerol | ^1^ |  | 1:400 |
| rabbit anti-phospho-Mad  (p-Smad 1/5) | Cell Signaling Technology | #41D10, [AB_491015](http://antibodyregistry.org/AB_491015) | 1:100-1:200 |
| mouse anti-dSRF | Cold Spring Harbor Laboratory Antibody Facility, gift from S. Blair |  | 1:300-1:400 |
| rabbit anti-vermiform | ^2^ |  | 1:300-1:400 |
| rat anti-DE-cadherin | DSHB | # DCAD2, [AB_528120](http://antibodyregistry.org/AB_528120) | 1:25 |
| rabbit mAb anti-phospho-SAPK/JNK (Thr183/Tyr185) (81E11) | Cell Signaling Technology | #4668 | 1:300 |
| mouse anti-phosphotyrosine, clone 4G10 | Sigma-Aldrich | 05-321 | 1:400 |
| secondary antibodies Alexa Fluor 488/ 568/647 coupled | Thermo Fisher Scientific |  | 1:500 |
|  |  |  |  |
| **Experimental Models: Cell Lines** | | |  |
| *D. melanogaster*: Stable S2 line expressing Sqh::GFP | ^3^ |  |  |
|  |  |  |  |
| **Experimental Models: Organisms/Strains** | | |  |
| *w; ; UAS_RhoKinase::dGBP1* |  |  |  |
| *w; enGal4 UAS_N-Rok::vhhGFP4^Vi^ /CyO DfdGMR_YFP* |  |  |  |
| *w; enGal4 UAS_mCherry-nls /(CyO DfdGMR_YFP)* |  |  |  |
| *(y) w; sqh_Sqh::GFP enGal4 UAS_mCherry-nls; +/(TM3)* |  |  |  |
| *w; enGal4 sqh_Sqh::mCherry* | *sqh_Sqh::mCherry:* ^4^ |  |  |
| *w; ; UAS_N-Rok::vhhGFP4^ZH-86Fb^* |  |  |  |
| *(y) w; UAS_N-Rok::vhhGFP4^Vi^ /P{2xTb[1]-RFP}CyO* | *P{2xTb[1]-RFP}CyO:* Bloomington #36336 |  |  |
| *w; ; UAS_ N-RokDead::vhhGFP4^ZH86Fb^* |  |  |  |
| *(y) w; ; UAS_ N-Rok::2m22^ZH-86Fb^* |  |  |  |
| *(y) w; ; UAS_ N-RokDead::2m22^ZH-86Fb^* |  |  |  |
| *w; ; UAS_ N-Rok-HA^ZH-86Fb^* |  |  |  |
| *w; ; UAS_vhhGFP4^ZH-86Fb^* |  |  |  |
| *w[1118]; ; UAS_ctMLCK/TM3 BL37527* | ^5^ |  |  |
| *w; ; UAS_DRok-cat T2A* | ^6^ |  |  |
| *y w; ; UAS_rokCAT BL6669* | ^7^ |  |  |
| *w; ; UAS_sqhEE/TM3 Ser* | ^7^ |  |  |
| *w; enGal4/TSTL; UAS_ N-RokDead::vhhGFP4^ZH-86Fb^/TSTL* |  |  |  |
| *yw* |  |  |  |
| *yw ; shg::GFP; + / TM3,Sb* | ^8^ |  |  |
| *y w ; {UAS::a-cat::mCherry} / TM3* |  |  |  |
| *sqh[AX3] ; {sqh_Sqh::GFP} {btl-Gal4} {UAS-mCherry-nls}* | *sqh_Sqh::GFP*:^9^ ; *btl-Gal4*: from Shigeo Hayashi; *UAS- mCherryn*ls: ^10^ |  |  |
| *Tkv::YFP* | Gift from G. Pyrowolakis |  |  |
| *#476 sqh[AX3]/(Fm7); {sqh_Sqh::GFP} {UAS-Lifeact::mRuby}* | *sqh_Sqh::GFP*:^9^ ; *UAS-Lifeact::Ruby*:^11^ |  |  |
| *{btl-Gal4}/CyO; UAS_RhoKinase::dGBP1/Tm3* | *btl-Gal4*: from Shigeo Hayashi |  |  |
| *{kni-Gal4} ; TM3 / TM6* | ^12^ |  |  |
| *UAS_bsk::GFP* | Bloomington #59267 Zeidler, M. (2015.2.13) |  |  |
| *enGal4 UAS_bsk::GFP* | *UAS_bsk::GFP (*Bloomington #59267) |  |  |
| *If / CyO; UAS_dGBP1-HA::Src Y400E / (Tm6 Tb Hu)* |  |  |  |
| *If / CyO; UAS_dGBP1-HA::Src Y400D / (Tm6 Tb Hu)* |  |  |  |
| *If / CyO; UAS_ dGBP1-HA::SrcDead / (Tm6 Tb Hu)* |  |  |  |
| *If / CyO; UAS_Src Y400E / (Tm6 Tb Hu)* |  |  |  |
| *If / CyO; UAS_Src Y400D / (Tm6 Tb Hu)* |  |  |  |
| *If / CyO; UAS_SrcDead / (Tm6 Tb Hu)* |  |  |  |
| *If / CyO; UAS_Src Y400E::dGBP1-HA / (Tm6 Tb Hu)* |  |  |  |
| *If / CyO; UAS_Src Y400D::dGBP1-HA / (Tm6 Tb Hu)* |  |  |  |
| *If / CyO; UAS_SrcDead::dGBP-HA / (Tm6 Tb Hu)* |  |  |  |
| *If / CyO; UAS_Src::dGBP1-HA / (Tm6 Tb Hu)* |  |  |  |
|  |  |  |  |
| **Plasmids** | | |  |
| pActin_Gal4 |  |  |  |
| pUASTattB | ^13^ |  |  |
| pUASTattB_N-Rok::vhhGFP4 |  |  |  |
| pUAST_N-Rok::vhhGFP4 |  |  |  |
| pUASTattB_N-Rok::vhhGFP4-HA |  |  |  |
| pUASattB_N-RokDead::vhhGFP4-HA |  |  |  |
| pUASTattB_N-Rok-HA |  |  |  |
| pUASattB_N-RokDead-HA |  |  |  |
| pUASTattB_N-Rok::dGBP1 |  |  |  |
| pUASTattB_N-Rok::2m22 |  |  |  |
| pUASTattB_N-RokDead::2m22 |  |  |  |

1. Zhang, L. & Ward 4th, R. E. Distinct tissue distributions and subcellular localizations of differently phosphorylated forms of the myosin regulatory light chain in Drosophila. *Gene Expr Patterns* **11**, 93–104 (2011).

2. Luschnig, S., Bätz, T., Armbruster, K. & Krasnow, M. A. serpentine and vermiform encode matrix proteins with chitin binding and deacetylation domains that limit tracheal tube length in Drosophila. *Curr. Biol. CB* **16**, 186–194 (2006).

3. Rogers, S. L., Wiedemann, U., Häcker, U., Turck, C. & Vale, R. D. Drosophila RhoGEF2 associates with microtubule plus ends in an EB1-dependent manner. *Curr. Biol.* **14**, 1827–1833 (2004).

4. Martin, A. C., Kaschube, M. & Wieschaus, E. F. Pulsed contractions of an actin-myosin network drive apical constriction. *Nature* **457**, 495–499 (2009).

5. Kim, Y.-S., Fritz, J. L., Seneviratne, A. K. & VanBerkum, M. F. A. Constitutively active myosin light chain kinase alters axon guidance decisions in Drosophila embryos. *Dev Biol* **249**, 367–381 (2002).

6. Verdier, V., Guang-Chao-Chen & Settleman, J. Rho-kinase regulates tissue morphogenesis via non-muscle myosin and LIM-kinase during Drosophila development. *BMC Dev Biol* **6**, 38 (2006).

7. Winter, C. G. *et al.* Drosophila Rho-associated kinase (Drok) links Frizzled-mediated planar cell polarity signaling to the actin cytoskeleton. *Cell* **105**, 81–91 (2001).

8. Huang, J., Zhou, W., Dong, W., Watson, A. M. & Hong, Y. Directed, efficient, and versatile modifications of the Drosophila genome by genomic engineering. *Proc. Natl. Acad. Sci. U. S. A.* **106**, 8284–8289 (2009).

9. Royou, A., Field, C., Sisson, J. C., Sullivan, W. & Karess, R. Reassessing the role and dynamics of nonmuscle myosin II during furrow formation in early Drosophila embryos. *Mol Biol Cell* **15**, 838–850 (2004).

10. Caussinus, E., Colombelli, J. & Affolter, M. Tip-Cell Migration Controls Stalk-Cell Intercalation during Drosophila Tracheal Tube Elongation. *Curr. Biol.* **18**, 1727–1734 (2008).

11. Hatan, M., Shinder, V., Israeli, D., Schnorrer, F. & Volk, T. The Drosophila blood brain barrier is maintained by GPCR-dependent dynamic actin structures. *J. Cell Biol.* **192**, 307–319 (2011).

12. Jaspers, M. H. J. *et al.* The fatty acyl-CoA reductase Waterproof mediates airway clearance in Drosophila. *Dev. Biol.* **385**, 23–31 (2014).

13. Bischof, J., Maeda, R. K., Hediger, M., Karch, F. & Basler, K. An optimized transgenesis system for Drosophila using germ-line-specific phiC31 integrases. *Proc Natl Acad Sci U A* **104**, 3312–3317 (2007).
