# Supplementary material for: Engineered kinases as a tool for phosphorylation of selected targets in vivo
Source: J Cell Biol. 2022 Sep 14;221(10):e202106179. doi: 10.1083/jcb.202106179 (PMC9477969; doi:10.1083/jcb.202106179)
Supplement: Data S1 — is a multi-FASTA file corresponding to all the inserts used in this work. [file JCB_202106179_DataS1.docx]

>N-Rok::vhhGFP4

gaattcaaa**ATGCCAGCTGGACGAGAAACTGTGACCAAGCAGCGCAGCATGGATGTGGAACGAAGGCGCCGGGCGAATACGCTCGAGCGCGAGATGCGCGATCCGACCAGCATCTGCAATGTGGACTGCCTGCTGGACACGGTGTCGGCCTTGGTCAGCGATTGTGACCACGAGTCCCTGAGGCGGCTTAAGAACATCGAGCAGTATGCGGCCAAATACAAACCATTGGCAATGCAGATCAATCAGCTGCGCATGAACGTCGAGGATTTCCATTTTATCAAACTCATCGGCGCCGGCGCCTTTGGAGAGGTGCAGCTGGTGCGGCACAAGTCCTCCAGCCAGGTGTACGCCATGAAGCGGCTGTCCAAATTCGAGATGATGAAGCGACCGGACTCCGCGTTCTTCTGGGAGGAGCGACACATTATGGCGCACGCCAACTCCGAATGGATCGTTCAGCTGCACTTTGCATTTCAGGATGCCAAATACCTATACATGGTGATGGACTTTATGCCCGGCGGCGATATAGTCTCGCTGATGGGTGACTACGACATCCCGGAGAAGTGGGCCATCTTTTACACAATGGAGGTGGTTCTCGCGCTGGACACCATTCACAATATGGGCTTCGTGCATCGGGACGTTAAGCCGGATAATATGCTCCTCGACAGCTATGGCCACCTGAAATTGGCCGACTTTGGCACCTGTATGCGAATGGGCGCCAATGGCCAAGTGGTGTCCAGCAATGCCGTTGGCACGCCGGACTACATCAGCCCGGAGGTGCTGCAGTCGCAGGGCGTGGACAACGAGTACGGGCGGGAATGCGACTGGTGGTCGGTGGGCATTTTCCTCTACGAGATGCTGTTCGGCGAGACACCATTCTACGCAGACTCGCTGGTGGGCACCTACGGCAAGATCATGGACCACAAGAACTCGCTTAGCTTTCCACCCGAGGTTGAGATCAGCGAACAGGCGAAGGCACTGATCCGCGCCTTTCTCACGGACCGAACGCAACGTTTGGGTCGCTACGGCATCGAGGACATTAAGGCGCATCCGTTTTTCCGAAATGATACCTGGTCGTTCGACAACATTCGCGAGAGTGTGCCGCCTGTGGTGCCCGAACTCTCCTCCGACGATGATACGCGAAATTTCGAGGACATTGAGCGCGATGAGAAGCCGGAGGAAGTGTTTCCCGTACCCAAGGGCTTCGATGGCAATCACTTGCCCTTCATCGGCTTTACGTATACGGGTGACTATCAGCTGCTGTCTAGCGACACCGTGGATGCGGAGTCGAAGGAGGCCAATGTGGCAAACAGCGGGGCGGCCAGCAATAATCACGGCCACGGTCATAATCACCGCCATCGGCCGTCG**caattcaaaATGGATCAAGTCCAACTGGTGGAGTCTGGTGGCGCTTTGGTGCAGCCAGGTGGCTCTCTGCGTTTGTCCTGTGCCGCTTCTGGCTTCCCAGTGAACCGCTATTCCATGCGCTGGTATCGCCAGGCTCCAGGCAAAGAGCGTGAGTGGGTAGCCGGTATGTCCAGCGCGGGTGATCGTAGCTCCTATGAAGACTCCGTGAAGGGCCGTTTCACCATCAGCCGTGACGATGCCCGTAACACGGTGTATCTGCAAATGAACAGCTTGAAACCTGAAGATACGGCCGTGTATTACTGTAATGTGAACGTGGGCTTCGAGTATTGGGGCCAAGGCACCCAGGTCACCGTCTCCAGCtaatctaga

>N-Rok::vhhGFP4-HA

gaattcaaa**ATGCCAGCTGGACGAGAAACTGTGACCAAGCAGCGCAGCATGGATGTGGAACGAAGGCGCCGGGCGAATACGCTCGAGCGCGAGATGCGCGATCCGACCAGCATCTGCAATGTGGACTGCCTGCTGGACACGGTGTCGGCCTTGGTCAGCGATTGTGACCACGAGTCCCTGAGGCGGCTTAAGAACATCGAGCAGTATGCGGCCAAATACAAACCATTGGCAATGCAGATCAATCAGCTGCGCATGAACGTCGAGGATTTCCATTTTATCAAACTCATCGGCGCCGGCGCCTTTGGAGAGGTGCAGCTGGTGCGGCACAAGTCCTCCAGCCAGGTGTACGCCATGAAGCGGCTGTCCAAATTCGAGATGATGAAGCGACCGGACTCCGCGTTCTTCTGGGAGGAGCGACACATTATGGCGCACGCCAACTCCGAATGGATCGTTCAGCTGCACTTTGCATTTCAGGATGCCAAATACCTATACATGGTGATGGACTTTATGCCCGGCGGCGATATAGTCTCGCTGATGGGTGACTACGACATCCCGGAGAAGTGGGCCATCTTTTACACAATGGAGGTGGTTCTCGCGCTGGACACCATTCACAATATGGGCTTCGTGCATCGGGACGTTAAGCCGGATAATATGCTCCTCGACAGCTATGGCCACCTGAAATTGGCCGACTTTGGCACCTGTATGCGAATGGGCGCCAATGGCCAAGTGGTGTCCAGCAATGCCGTTGGCACGCCGGACTACATCAGCCCGGAGGTGCTGCAGTCGCAGGGCGTGGACAACGAGTACGGGCGGGAATGCGACTGGTGGTCGGTGGGCATTTTCCTCTACGAGATGCTGTTCGGCGAGACACCATTCTACGCAGACTCGCTGGTGGGCACCTACGGCAAGATCATGGACCACAAGAACTCGCTTAGCTTTCCACCCGAGGTTGAGATCAGCGAACAGGCGAAGGCACTGATCCGCGCCTTTCTCACGGACCGAACGCAACGTTTGGGTCGCTACGGCATCGAGGACATTAAGGCGCATCCGTTTTTCCGAAATGATACCTGGTCGTTCGACAACATTCGCGAGAGTGTGCCGCCTGTGGTGCCCGAACTCTCCTCCGACGATGATACGCGAAATTTCGAGGACATTGAGCGCGATGAGAAGCCGGAGGAAGTGTTTCCCGTACCCAAGGGCTTCGATGGCAATCACTTGCCCTTCATCGGCTTTACGTATACGGGTGACTATCAGCTGCTGTCTAGCGACACCGTGGATGCGGAGTCGAAGGAGGCCAATGTGGCAAACAGCGGGGCGGCCAGCAATAATCACGGCCACGGTCATAATCACCGCCATCGGCCGTCG**ctcgagATGGACCAGGTGCAGCTGGTGGAGTCCGGCGGCGCCCTGGTGCAGCCCGGCGGCTCCCTGCGCCTGTCCTGCGCCGCCTCCGGCTTCCCCGTGAACCGCTACTCCATGCGCTGGTACCGCCAGGCCCCCGGCAAGGAGCGCGAGTGGGTGGCCGGCATGTCCTCCGCCGGCGACCGCTCCTCCTACGAGGACTCCGTGAAGGGCCGCTTCACCATCTCCCGCGACGACGCCCGCAACACCGTGTACCTGCAGATGAACTCCCTGAAGCCCGAGGACACCGCCGTGTACTACTGCAACGTGAACGTGGGCTTCGAGTACTGGGGCCAGGGCACCCAGGTGACCGTGTCCTCCTACCCCTACGACGTGCCCGACTACGCCTAAaccggtaaatctaga

>N-RokDead::vhhGFP4-HA

gaattcaaa**ATGCCAGCTGGACGAGAAACTGTGACCAAGCAGCGCAGCATGGATGTGGAACGAAGGCGCCGGGCGAATACGCTCGAGCGCGAGATGCGCGATCCGACCAGCATCTGCAATGTGGACTGCCTGCTGGACACGGTGTCGGCCTTGGTCAGCGATTGTGACCACGAGTCCCTGAGGCGGCTTAAGAACATCGAGCAGTATGCGGCCAAATACAAACCATTGGCAATGCAGATCAATCAGCTGCGCATGAACGTCGAGGATTTCCATTTTATCAAACTCATCGGCGCCGGCGCCTTTGGAGAGGTGCAGCTGGTGCGGCACAAGTCCTCCAGCCAGGTGTACGCCATGGGGCGGCTGTCCAAATTCGAGATGATGAAGCGACCGGACTCCGCGTTCTTCTGGGAGGAGCGACACATTATGGCGCACGCCAACTCCGAATGGATCGTTCAGCTGCACTTTGCATTTCAGGATGCCAAATACCTATACATGGTGATGGACTTTATGCCCGGCGGCGATATAGTCTCGCTGATGGGTGACTACGACATCCCGGAGAAGTGGGCCATCTTTTACACAATGGAGGTGGTTCTCGCGCTGGACACCATTCACAATATGGGCTTCGTGCATCGGGACGTTAAGCCGGATAATATGCTCCTCGACAGCTATGGCCACCTGAAATTGGCCGACTTTGGCACCTGTATGCGAATGGGCGCCAATGGCCAAGTGGTGTCCAGCAATGCCGTTGGCACGCCGGACTACATCAGCCCGGAGGTGCTGCAGTCGCAGGGCGTGGACAACGAGTACGGGCGGGAATGCGACTGGTGGTCGGTGGGCATTTTCCTCTACGAGATGCTGTTCGGCGAGACACCATTCTACGCAGACTCGCTGGTGGGCACCTACGGCAAGATCATGGACCACAAGAACTCGCTTAGCTTTCCACCCGAGGTTGAGATCAGCGAACAGGCGAAGGCACTGATCCGCGCCTTTCTCACGGACCGAACGCAACGTTTGGGTCGCTACGGCATCGAGGACATTAAGGCGCATCCGTTTTTCCGAAATGATACCTGGTCGTTCGACAACATTCGCGAGAGTGTGCCGCCTGTGGTGCCCGAACTCTCCTCCGACGATGATACGCGAAATTTCGAGGACATTGAGCGCGATGAGAAGCCGGAGGAAGTGTTTCCCGTACCCAAGGGCTTCGATGGCAATCACTTGCCCTTCATCGGCTTTACGTATACGGGTGACTATCAGCTGCTGTCTAGCGACACCGTGGATGCGGAGTCGAAGGAGGCCAATGTGGCAAACAGCGGGGCGGCCAGCAATAATCACGGCCACGGTCATAATCACCGCCATCGGCCGTCG**ctcgagATGGACCAGGTGCAGCTGGTGGAGTCCGGCGGCGCCCTGGTGCAGCCCGGCGGCTCCCTGCGCCTGTCCTGCGCCGCCTCCGGCTTCCCCGTGAACCGCTACTCCATGCGCTGGTACCGCCAGGCCCCCGGCAAGGAGCGCGAGTGGGTGGCCGGCATGTCCTCCGCCGGCGACCGCTCCTCCTACGAGGACTCCGTGAAGGGCCGCTTCACCATCTCCCGCGACGACGCCCGCAACACCGTGTACCTGCAGATGAACTCCCTGAAGCCCGAGGACACCGCCGTGTACTACTGCAACGTGAACGTGGGCTTCGAGTACTGGGGCCAGGGCACCCAGGTGACCGTGTCCTCCTACCCCTACGACGTGCCCGACTACGCCataaccggtaaatctaga

>N-Rok-HA

gaattcaaa**ATGCCAGCTGGACGAGAAACTGTGACCAAGCAGCGCAGCATGGATGTGGAACGAAGGCGCCGGGCGAATACGCTCGAGCGCGAGATGCGCGATCCGACCAGCATCTGCAATGTGGACTGCCTGCTGGACACGGTGTCGGCCTTGGTCAGCGATTGTGACCACGAGTCCCTGAGGCGGCTTAAGAACATCGAGCAGTATGCGGCCAAATACAAACCATTGGCAATGCAGATCAATCAGCTGCGCATGAACGTCGAGGATTTCCATTTTATCAAACTCATCGGCGCCGGCGCCTTTGGAGAGGTGCAGCTGGTGCGGCACAAGTCCTCCAGCCAGGTGTACGCCATGAAGCGGCTGTCCAAATTCGAGATGATGAAGCGACCGGACTCCGCGTTCTTCTGGGAGGAGCGACACATTATGGCGCACGCCAACTCCGAATGGATCGTTCAGCTGCACTTTGCATTTCAGGATGCCAAATACCTATACATGGTGATGGACTTTATGCCCGGCGGCGATATAGTCTCGCTGATGGGTGACTACGACATCCCGGAGAAGTGGGCCATCTTTTACACAATGGAGGTGGTTCTCGCGCTGGACACCATTCACAATATGGGCTTCGTGCATCGGGACGTTAAGCCGGATAATATGCTCCTCGACAGCTATGGCCACCTGAAATTGGCCGACTTTGGCACCTGTATGCGAATGGGCGCCAATGGCCAAGTGGTGTCCAGCAATGCCGTTGGCACGCCGGACTACATCAGCCCGGAGGTGCTGCAGTCGCAGGGCGTGGACAACGAGTACGGGCGGGAATGCGACTGGTGGTCGGTGGGCATTTTCCTCTACGAGATGCTGTTCGGCGAGACACCATTCTACGCAGACTCGCTGGTGGGCACCTACGGCAAGATCATGGACCACAAGAACTCGCTTAGCTTTCCACCCGAGGTTGAGATCAGCGAACAGGCGAAGGCACTGATCCGCGCCTTTCTCACGGACCGAACGCAACGTTTGGGTCGCTACGGCATCGAGGACATTAAGGCGCATCCGTTTTTCCGAAATGATACCTGGTCGTTCGACAACATTCGCGAGAGTGTGCCGCCTGTGGTGCCCGAACTCTCCTCCGACGATGATACGCGAAATTTCGAGGACATTGAGCGCGATGAGAAGCCGGAGGAAGTGTTTCCCGTACCCAAGGGCTTCGATGGCAATCACTTGCCCTTCATCGGCTTTACGTATACGGGTGACTATCAGCTGCTGTCTAGCGACACCGTGGATGCGGAGTCGAAGGAGGCCAATGTGGCAAACAGCGGGGCGGCCAGCAATAATCACGGCCACGGTCATAATCACCGCCATCGGCCGTCG**ctcgagTACCCCTACGACGTGCCCGACTACGCCtaatctaga

>N-RokDead-HA

**ATGCCAGCTGGACGAGAAACTGTGACCAAGCAGCGCAGCATGGATGTGGAACGAAGGCGCCGGGCGAATACGCTCGAGCGCGAGATGCGCGATCCGACCAGCATCTGCAATGTGGACTGCCTGCTGGACACGGTGTCGGCCTTGGTCAGCGATTGTGACCACGAGTCCCTGAGGCGGCTTAAGAACATCGAGCAGTATGCGGCCAAATACAAACCATTGGCAATGCAGATCAATCAGCTGCGCATGAACGTCGAGGATTTCCATTTTATCAAACTCATCGGCGCCGGCGCCTTTGGAGAGGTGCAGCTGGTGCGGCACAAGTCCTCCAGCCAGGTGTACGCCATGGGGCGGCTGTCCAAATTCGAGATGATGAAGCGACCGGACTCCGCGTTCTTCTGGGAGGAGCGACACATTATGGCGCACGCCAACTCCGAATGGATCGTTCAGCTGCACTTTGCATTTCAGGATGCCAAATACCTATACATGGTGATGGACTTTATGCCCGGCGGCGATATAGTCTCGCTGATGGGTGACTACGACATCCCGGAGAAGTGGGCCATCTTTTACACAATGGAGGTGGTTCTCGCGCTGGACACCATTCACAATATGGGCTTCGTGCATCGGGACGTTAAGCCGGATAATATGCTCCTCGACAGCTATGGCCACCTGAAATTGGCCGACTTTGGCACCTGTATGCGAATGGGCGCCAATGGCCAAGTGGTGTCCAGCAATGCCGTTGGCACGCCGGACTACATCAGCCCGGAGGTGCTGCAGTCGCAGGGCGTGGACAACGAGTACGGGCGGGAATGCGACTGGTGGTCGGTGGGCATTTTCCTCTACGAGATGCTGTTCGGCGAGACACCATTCTACGCAGACTCGCTGGTGGGCACCTACGGCAAGATCATGGACCACAAGAACTCGCTTAGCTTTCCACCCGAGGTTGAGATCAGCGAACAGGCGAAGGCACTGATCCGCGCCTTTCTCACGGACCGAACGCAACGTTTGGGTCGCTACGGCATCGAGGACATTAAGGCGCATCCGTTTTTCCGAAATGATACCTGGTCGTTCGACAACATTCGCGAGAGTGTGCCGCCTGTGGTGCCCGAACTCTCCTCCGACGATGATACGCGAAATTTCGAGGACATTGAGCGCGATGAGAAGCCGGAGGAAGTGTTTCCCGTACCCAAGGGCTTCGATGGCAATCACTTGCCCTTCATCGGCTTTACGTATACGGGTGACTATCAGCTGCTGTCTAGCGACACCGTGGATGCGGAGTCGAAGGAGGCCAATGTGGCAAACAGCGGGGCGGCCAGCAATAATCACGGCCACGGTCATAATCACCGCCATCGGCCGTCG**ctcgagTACCCCTACGACGTGCCCGACTACGCCtaatctaga

>N-Rok::2m22

gaattcaaa**ATGCCAGCTGGACGAGAAACTGTGACCAAGCAGCGCAGCATGGATGTGGAACGAAGGCGCCGGGCGAATACGCTCGAGCGCGAGATGCGCGATCCGACCAGCATCTGCAATGTGGACTGCCTGCTGGACACGGTGTCGGCCTTGGTCAGCGATTGTGACCACGAGTCCCTGAGGCGGCTTAAGAACATCGAGCAGTATGCGGCCAAATACAAACCATTGGCAATGCAGATCAATCAGCTGCGCATGAACGTCGAGGATTTCCATTTTATCAAACTCATCGGCGCCGGCGCCTTTGGAGAGGTGCAGCTGGTGCGGCACAAGTCCTCCAGCCAGGTGTACGCCATGAAGCGGCTGTCCAAATTCGAGATGATGAAGCGACCGGACTCCGCGTTCTTCTGGGAGGAGCGACACATTATGGCGCACGCCAACTCCGAATGGATCGTTCAGCTGCACTTTGCATTTCAGGATGCCAAATACCTATACATGGTGATGGACTTTATGCCCGGCGGCGATATAGTCTCGCTGATGGGTGACTACGACATCCCGGAGAAGTGGGCCATCTTTTACACAATGGAGGTGGTTCTCGCGCTGGACACCATTCACAATATGGGCTTCGTGCATCGGGACGTTAAGCCGGATAATATGCTCCTCGACAGCTATGGCCACCTGAAATTGGCCGACTTTGGCACCTGTATGCGAATGGGCGCCAATGGCCAAGTGGTGTCCAGCAATGCCGTTGGCACGCCGGACTACATCAGCCCGGAGGTGCTGCAGTCGCAGGGCGTGGACAACGAGTACGGGCGGGAATGCGACTGGTGGTCGGTGGGCATTTTCCTCTACGAGATGCTGTTCGGCGAGACACCATTCTACGCAGACTCGCTGGTGGGCACCTACGGCAAGATCATGGACCACAAGAACTCGCTTAGCTTTCCACCCGAGGTTGAGATCAGCGAACAGGCGAAGGCACTGATCCGCGCCTTTCTCACGGACCGAACGCAACGTTTGGGTCGCTACGGCATCGAGGACATTAAGGCGCATCCGTTTTTCCGAAATGATACCTGGTCGTTCGACAACATTCGCGAGAGTGTGCCGCCTGTGGTGCCCGAACTCTCCTCCGACGATGATACGCGAAATTTCGAGGACATTGAGCGCGATGAGAAGCCGGAGGAAGTGTTTCCCGTACCCAAGGGCTTCGATGGCAATCACTTGCCCTTCATCGGCTTTACGTATACGGGTGACTATCAGCTGCTGTCTAGCGACACCGTGGATGCGGAGTCGAAGGAGGCCAATGTGGCAAACAGCGGGGCGGCCAGCAATAATCACGGCCACGGTCATAATCACCGCCATCGGCCGTCG**ctcgagATGAGAGGATCGCATCACCATCACCATCACGGATCCGACCTGGGTAAGAAACTGCTGGAAGCTGCTCGTGTTGGTCAGGACGACGAAGTTCGTATCCTGATGGCTAACGGTGCTGACGTTAACGCTTATGACACTATTGGTTCTACTCCGCTGCACCTGGCTGCTTATCATGGTCACCTGGAAATCGTTGAAGTTCTGCTGAAGAACGGTGCTGACGTTAACGCTAATGACCTTTTTGGTGATACTCCGCTGCACCTGGCTGCTTATTCTGGTCACCTGGAAATCGTTGAAGTTCTGCTGAAGTACGGTGCTGACGTTAACGCTCAGGACAAATTCGGTAAGACCGCTTTCGACATCTCCATCGACAACGGTAACGAGGACCTGGCTGAAATCCTGCAAAAGCTTAATtaatctaga

>N-RokDead::2m22

gaattcaaa**ATGCCAGCTGGACGAGAAACTGTGACCAAGCAGCGCAGCATGGATGTGGAACGAAGGCGCCGGGCGAATACGCTCGAGCGCGAGATGCGCGATCCGACCAGCATCTGCAATGTGGACTGCCTGCTGGACACGGTGTCGGCCTTGGTCAGCGATTGTGACCACGAGTCCCTGAGGCGGCTTAAGAACATCGAGCAGTATGCGGCCAAATACAAACCATTGGCAATGCAGATCAATCAGCTGCGCATGAACGTCGAGGATTTCCATTTTATCAAACTCATCGGCGCCGGCGCCTTTGGAGAGGTGCAGCTGGTGCGGCACAAGTCCTCCAGCCAGGTGTACGCCATGGGGCGGCTGTCCAAATTCGAGATGATGAAGCGACCGGACTCCGCGTTCTTCTGGGAGGAGCGACACATTATGGCGCACGCCAACTCCGAATGGATCGTTCAGCTGCACTTTGCATTTCAGGATGCCAAATACCTATACATGGTGATGGACTTTATGCCCGGCGGCGATATAGTCTCGCTGATGGGTGACTACGACATCCCGGAGAAGTGGGCCATCTTTTACACAATGGAGGTGGTTCTCGCGCTGGACACCATTCACAATATGGGCTTCGTGCATCGGGACGTTAAGCCGGATAATATGCTCCTCGACAGCTATGGCCACCTGAAATTGGCCGACTTTGGCACCTGTATGCGAATGGGCGCCAATGGCCAAGTGGTGTCCAGCAATGCCGTTGGCACGCCGGACTACATCAGCCCGGAGGTGCTGCAGTCGCAGGGCGTGGACAACGAGTACGGGCGGGAATGCGACTGGTGGTCGGTGGGCATTTTCCTCTACGAGATGCTGTTCGGCGAGACACCATTCTACGCAGACTCGCTGGTGGGCACCTACGGCAAGATCATGGACCACAAGAACTCGCTTAGCTTTCCACCCGAGGTTGAGATCAGCGAACAGGCGAAGGCACTGATCCGCGCCTTTCTCACGGACCGAACGCAACGTTTGGGTCGCTACGGCATCGAGGACATTAAGGCGCATCCGTTTTTCCGAAATGATACCTGGTCGTTCGACAACATTCGCGAGAGTGTGCCGCCTGTGGTGCCCGAACTCTCCTCCGACGATGATACGCGAAATTTCGAGGACATTGAGCGCGATGAGAAGCCGGAGGAAGTGTTTCCCGTACCCAAGGGCTTCGATGGCAATCACTTGCCCTTCATCGGCTTTACGTATACGGGTGACTATCAGCTGCTGTCTAGCGACACCGTGGATGCGGAGTCGAAGGAGGCCAATGTGGCAAACAGCGGGGCGGCCAGCAATAATCACGGCCACGGTCATAATCACCGCCATCGGCCGTCG**ctcgagATGAGAGGATCGCATCACCATCACCATCACGGATCCGACCTGGGTAAGAAACTGCTGGAAGCTGCTCGTGTTGGTCAGGACGACGAAGTTCGTATCCTGATGGCTAACGGTGCTGACGTTAACGCTTATGACACTATTGGTTCTACTCCGCTGCACCTGGCTGCTTATCATGGTCACCTGGAAATCGTTGAAGTTCTGCTGAAGAACGGTGCTGACGTTAACGCTAATGACCTTTTTGGTGATACTCCGCTGCACCTGGCTGCTTATTCTGGTCACCTGGAAATCGTTGAAGTTCTGCTGAAGTACGGTGCTGACGTTAACGCTCAGGACAAATTCGGTAAGACCGCTTTCGACATCTCCATCGACAACGGTAACGAGGACCTGGCTGAAATCCTGCAAAAGCTTAATtaatctaga

>N-Rok::dGBP1-HA

gaattcaaa**ATGCCAGCTGGACGAGAAACTGTGACCAAGCAGCGCAGCATGGATGTGGAACGAAGGCGCCGGGCGAATACGCTCGAGCGCGAGATGCGCGATCCGACCAGCATCTGCAATGTGGACTGCCTGCTGGACACGGTGTCGGCCTTGGTCAGCGATTGTGACCACGAGTCCCTGAGGCGGCTTAAGAACATCGAGCAGTATGCGGCCAAATACAAACCATTGGCAATGCAGATCAATCAGCTGCGCATGAACGTCGAGGATTTCCATTTTATCAAACTCATCGGCGCCGGCGCCTTTGGAGAGGTGCAGCTGGTGCGGCACAAGTCCTCCAGCCAGGTGTACGCCATGAAGCGGCTGTCCAAATTCGAGATGATGAAGCGACCGGACTCCGCGTTCTTCTGGGAGGAGCGACACATTATGGCGCACGCCAACTCCGAATGGATCGTTCAGCTGCACTTTGCATTTCAGGATGCCAAATACCTATACATGGTGATGGACTTTATGCCCGGCGGCGATATAGTCTCGCTGATGGGTGACTACGACATCCCGGAGAAGTGGGCCATCTTTTACACAATGGAGGTGGTTCTCGCGCTGGACACCATTCACAATATGGGCTTCGTGCATCGGGACGTTAAGCCGGATAATATGCTCCTCGACAGCTATGGCCACCTGAAATTGGCCGACTTTGGCACCTGTATGCGAATGGGCGCCAATGGCCAAGTGGTGTCCAGCAATGCCGTTGGCACGCCGGACTACATCAGCCCGGAGGTGCTGCAGTCGCAGGGCGTGGACAACGAGTACGGGCGGGAATGCGACTGGTGGTCGGTGGGCATTTTCCTCTACGAGATGCTGTTCGGCGAGACACCATTCTACGCAGACTCGCTGGTGGGCACCTACGGCAAGATCATGGACCACAAGAACTCGCTTAGCTTTCCACCCGAGGTTGAGATCAGCGAACAGGCGAAGGCACTGATCCGCGCCTTTCTCACGGACCGAACGCAACGTTTGGGTCGCTACGGCATCGAGGACATTAAGGCGCATCCGTTTTTCCGAAATGATACCTGGTCGTTCGACAACATTCGCGAGAGTGTGCCGCCTGTGGTGCCCGAACTCTCCTCCGACGATGATACGCGAAATTTCGAGGACATTGAGCGCGATGAGAAGCCGGAGGAAGTGTTTCCCGTACCCAAGGGCTTCGATGGCAATCACTTGCCCTTCATCGGCTTTACGTATACGGGTGACTATCAGCTGCTGTCTAGCGACACCGTGGATGCGGAGTCGAAGGAGGCCAATGTGGCAAACAGCGGGGCGGCCAGCAATAATCACGGCCACGGTCATAATCACCGCCATCGGCCGTCG**ctcgagATGGCCGACGTGCAGCTCGTGGAATCCGGAGGAGCACTGGTCCAGCCTGGGGGATCACTGAGACTGTCTTGTGTCGCTTCTGGCTTTCCTGTGAATCGGTACTCTATGAGATGGTACAGGCAGGCACCTGGAAAAGAGCGAGAATGGGTCGCCGGAATGTCATCTGCTGGCGATAGGAGTTCCTACGTGGATAGCGTGAAAGGACGGTTCACCATTAGAAGGGACGATGCTAGAAACACCGTGTACCTCCAGATGAACTCCCTGAAACCTGAGGACACTGCTGTGTACTACTACAACGTCAATGTGGGCTTTGAATACTGGGGACATGGCACACAAGTGACTGTTAGTTTTTACCCCTACGACGTGCCCGACTACGCCtaaaccggtaaatctaga

>dGBP1-HA::SrcDead

gAATTCATGGCCGACGTGCAGCTCGTGGAATCCGGAGGAGCACTGGTCCAGCCTGGGGGATCACTGAGACTGTCTTGTGTCGCTTCTGGCTTTCCTGTGAATCGGTACTCTATGAGATGGTACAGGCAGGCACCTGGAAAAGAGCGAGAATGGGTCGCCGGAATGTCATCTGCTGGCGATAGGAGTTCCTACGTGGATAGCGTGAAAGGACGGTTCACCATTAGAAGGGACGATGCTAGAAACACCGTGTACCTCCAGATGAACTCCCTGAAACCTGAGGACACTGCTGTGTACTACTACAACGTCAATGTGGGCTTTGAATACTGGGGACATGGCACACAAGTGACTGTTAGTTTTTACCCCTACGACGTGCCCGACTACGCCGGCGGTGGAAGCGGCGGCGGATCCCTCGAG**CTATGCGTCAACCTCTGCAAGCCGTGTGTCCAGATCGAGAAGCCTGTAACTGAGGGGCTTTCGCACCGCACTCGCGATCAGTGGGAGATCGACAGAACGTCTTTGAAATTCGTGCGCAAACTGGGCTCCGGACAGTTTGGCGATGTCTGGGAGGGATTGTGGAACAACACAACACCTGTGGCAATTATGACTCTGAAATCTGGTACAATGGACCCCAAGGATTTCTTAGCGGAAGCCCAGATCATGAAGAAACTGCGCCACACCAAGCTTATACAGTTGTACGCTGTCTGCACTGTTGAGGAGCCTATCTATATTATCACAGAGTTAATGAAGCACGGTTCACTGTTGGAATATCTCCAAGCCATTGCAGGCAAGGGTCGTAGCCTTAAAATGCAAACTCTGATTGATATGGCAGCGCAAATAGCTGCTGGCATGGCTTACTTGGAGTCCCAGAATTATATTCATAGGGATTTAGCGGCGCGCAATGTACTGGTAGGCGATGGAAACATCGTCAAAATCGCCGACTTTGGTTTAGCTAGGCTCATCAAGGAGGACGAATACGAGGCGCGGGTAGGCGCCAGATTTCCCATAAAATGGACCGCTCCAGAGGCTGCTAACTACAGCAAATTCTCAATAAAATCGGATGTTTGGAGCTTTGGCATTCTTCTCACAGAACTGGTCACCTACGGACGCATACCATATCCAGGCATGACCAACGCTGAGGTGCTAACGCAAGTGGAGCACGGCTATCGAATGCCGCAACCTCCCAACTGCGAGCCGCGCCTGTATGAGATTATGCTGGAATGTTGGCACAAGGACCCCATGCGCAGACCCACGTTTGAGACGCTACAATGGAAACTGGAAGACTTCTATACATCTGATCAGAGCGACTtCAAAGAGGCGCAGGCCTACTGA**ttctagagga

>dGBP1-HA::Src Y400E

gAATTCATGGCCGACGTGCAGCTCGTGGAATCCGGAGGAGCACTGGTCCAGCCTGGGGGATCACTGAGACTGTCTTGTGTCGCTTCTGGCTTTCCTGTGAATCGGTACTCTATGAGATGGTACAGGCAGGCACCTGGAAAAGAGCGAGAATGGGTCGCCGGAATGTCATCTGCTGGCGATAGGAGTTCCTACGTGGATAGCGTGAAAGGACGGTTCACCATTAGAAGGGACGATGCTAGAAACACCGTGTACCTCCAGATGAACTCCCTGAAACCTGAGGACACTGCTGTGTACTACTACAACGTCAATGTGGGCTTTGAATACTGGGGACATGGCACACAAGTGACTGTTAGTTTTTACCCCTACGACGTGCCCGACTACGCCGGCGGTGGAAGCGGCGGCGGATCCCTCGAG**CTATGCGTCAACCTCTGCAAGCCGTGTGTCCAGATCGAGAAGCCTGTAACTGAGGGGCTTTCGCACCGCACTCGCGATCAGTGGGAGATCGACAGAACGTCTTTGAAATTCGTGCGCAAACTGGGCTCCGGACAGTTTGGCGATGTCTGGGAGGGATTGTGGAACAACACAACACCTGTGGCAATTAAAACTCTGAAATCTGGTACAATGGACCCCAAGGATTTCTTAGCGGAAGCCCAGATCATGAAGAAACTGCGCCACACCAAGCTTATACAGTTGTACGCTGTCTGCACTGTTGAGGAGCCTATCTATATTATCACAGAGTTAATGAAGCACGGTTCACTGTTGGAATATCTCCAAGCCATTGCAGGCAAGGGTCGTAGCCTTAAAATGCAAACTCTGATTGATATGGCAGCGCAAATAGCTGCTGGCATGGCTTACTTGGAGTCCCAGAATTATATTCATAGGGATTTAGCGGCGCGCAATGTACTGGTAGGCGATGGAAACATCGTCAAAATCGCCGACTTTGGTTTAGCTAGGCTCATCAAGGAGGACGAAGAAGAGGCGCGGGTAGGCGCCAGATTTCCCATAAAATGGACCGCTCCAGAGGCTGCTAACTACAGCAAATTCTCAATAAAATCGGATGTTTGGAGCTTTGGCATTCTTCTCACAGAACTGGTCACCTACGGACGCATACCATATCCAGGCATGACCAACGCTGAGGTGCTAACGCAAGTGGAGCACGGCTATCGAATGCCGCAACCTCCCAACTGCGAGCCGCGCCTGTATGAGATTATGCTGGAATGTTGGCACAAGGACCCCATGCGCAGACCCACGTTTGAGACGCTACAATGGAAACTGGAAGACTTCTATACATCTGATCAGAGCGACTtCAAAGAGGCGCAGGCCTACTGA**ttctagagga

>dGBP1-HA::Src Y400D

gAATTCATGGCCGACGTGCAGCTCGTGGAATCCGGAGGAGCACTGGTCCAGCCTGGGGGATCACTGAGACTGTCTTGTGTCGCTTCTGGCTTTCCTGTGAATCGGTACTCTATGAGATGGTACAGGCAGGCACCTGGAAAAGAGCGAGAATGGGTCGCCGGAATGTCATCTGCTGGCGATAGGAGTTCCTACGTGGATAGCGTGAAAGGACGGTTCACCATTAGAAGGGACGATGCTAGAAACACCGTGTACCTCCAGATGAACTCCCTGAAACCTGAGGACACTGCTGTGTACTACTACAACGTCAATGTGGGCTTTGAATACTGGGGACATGGCACACAAGTGACTGTTAGTTTTTACCCCTACGACGTGCCCGACTACGCCGGCGGTGGAAGCGGCGGCGGATCCCTCGAG**CTATGCGTCAACCTCTGCAAGCCGTGTGTCCAGATCGAGAAGCCTGTAACTGAGGGGCTTTCGCACCGCACTCGCGATCAGTGGGAGATCGACAGAACGTCTTTGAAATTCGTGCGCAAACTGGGCTCCGGACAGTTTGGCGATGTCTGGGAGGGATTGTGGAACAACACAACACCTGTGGCAATTAAAACTCTGAAATCTGGTACAATGGACCCCAAGGATTTCTTAGCGGAAGCCCAGATCATGAAGAAACTGCGCCACACCAAGCTTATACAGTTGTACGCTGTCTGCACTGTTGAGGAGCCTATCTATATTATCACAGAGTTAATGAAGCACGGTTCACTGTTGGAATATCTCCAAGCCATTGCAGGCAAGGGTCGTAGCCTTAAAATGCAAACTCTGATTGATATGGCAGCGCAAATAGCTGCTGGCATGGCTTACTTGGAGTCCCAGAATTATATTCATAGGGATTTAGCGGCGCGCAATGTACTGGTAGGCGATGGAAACATCGTCAAAATCGCCGACTTTGGTTTAGCTAGGCTCATCAAGGAGGACGAAGACGAGGCGCGGGTAGGCGCCAGATTTCCCATAAAATGGACCGCTCCAGAGGCTGCTAACTACAGCAAATTCTCAATAAAATCGGATGTTTGGAGCTTTGGCATTCTTCTCACAGAACTGGTCACCTACGGACGCATACCATATCCAGGCATGACCAACGCTGAGGTGCTAACGCAAGTGGAGCACGGCTATCGAATGCCGCAACCTCCCAACTGCGAGCCGCGCCTGTATGAGATTATGCTGGAATGTTGGCACAAGGACCCCATGCGCAGACCCACGTTTGAGACGCTACAATGGAAACTGGAAGACTTCTATACATCTGATCAGAGCGACTtCAAAGAGGCGCAGGCCTACTGA**ttctagagga

>SrcDead

taccTCGAG**ATGCTATGCGTCAACCTCTGCAAGCCGTGTGTCCAGATCGAGAAGCCTGTAACTGAGGGGCTTTCGCACCGCACTCGCGATCAGTGGGAGATCGACAGAACGTCTTTGAAATTCGTGCGCAAACTGGGCTCCGGACAGTTTGGCGATGTCTGGGAGGGATTGTGGAACAACACAACACCTGTGGCAATTATGACTCTGAAATCTGGTACAATGGACCCCAAGGATTTCTTAGCGGAAGCCCAGATCATGAAGAAACTGCGCCACACCAAGCTTATACAGTTGTACGCTGTCTGCACTGTTGAGGAGCCTATCTATATTATCACAGAGTTAATGAAGCACGGTTCACTGTTGGAATATCTCCAAGCCATTGCAGGCAAGGGTCGTAGCCTTAAAATGCAAACTCTGATTGATATGGCAGCGCAAATAGCTGCTGGCATGGCTTACTTGGAGTCCCAGAATTATATTCATAGGGATTTAGCGGCGCGCAATGTACTGGTAGGCGATGGAAACATCGTCAAAATCGCCGACTTTGGTTTAGCTAGGCTCATCAAGGAGGACGAATACGAGGCGCGGGTAGGCGCCAGATTTCCCATAAAATGGACCGCTCCAGAGGCTGCTAACTACAGCAAATTCTCAATAAAATCGGATGTTTGGAGCTTTGGCATTCTTCTCACAGAACTGGTCACCTACGGACGCATACCATATCCAGGCATGACCAACGCTGAGGTGCTAACGCAAGTGGAGCACGGCTATCGAATGCCGCAACCTCCCAACTGCGAGCCGCGCCTGTATGAGATTATGCTGGAATGTTGGCACAAGGACCCCATGCGCAGACCCACGTTTGAGACGCTACAATGGAAACTGGAAGACTTCTATACATCTGATCAGAGCGACTtCAAAGAGGCGCAGGCCTACTGA**ttctagagga

>Src Y400E

taccTCGAG**ATGCTATGCGTCAACCTCTGCAAGCCGTGTGTCCAGATCGAGAAGCCTGTAACTGAGGGGCTTTCGCACCGCACTCGCGATCAGTGGGAGATCGACAGAACGTCTTTGAAATTCGTGCGCAAACTGGGCTCCGGACAGTTTGGCGATGTCTGGGAGGGATTGTGGAACAACACAACACCTGTGGCAATTAAAACTCTGAAATCTGGTACAATGGACCCCAAGGATTTCTTAGCGGAAGCCCAGATCATGAAGAAACTGCGCCACACCAAGCTTATACAGTTGTACGCTGTCTGCACTGTTGAGGAGCCTATCTATATTATCACAGAGTTAATGAAGCACGGTTCACTGTTGGAATATCTCCAAGCCATTGCAGGCAAGGGTCGTAGCCTTAAAATGCAAACTCTGATTGATATGGCAGCGCAAATAGCTGCTGGCATGGCTTACTTGGAGTCCCAGAATTATATTCATAGGGATTTAGCGGCGCGCAATGTACTGGTAGGCGATGGAAACATCGTCAAAATCGCCGACTTTGGTTTAGCTAGGCTCATCAAGGAGGACGAAGAAGAGGCGCGGGTAGGCGCCAGATTTCCCATAAAATGGACCGCTCCAGAGGCTGCTAACTACAGCAAATTCTCAATAAAATCGGATGTTTGGAGCTTTGGCATTCTTCTCACAGAACTGGTCACCTACGGACGCATACCATATCCAGGCATGACCAACGCTGAGGTGCTAACGCAAGTGGAGCACGGCTATCGAATGCCGCAACCTCCCAACTGCGAGCCGCGCCTGTATGAGATTATGCTGGAATGTTGGCACAAGGACCCCATGCGCAGACCCACGTTTGAGACGCTACAATGGAAACTGGAAGACTTCTATACATCTGATCAGAGCGACTtCAAAGAGGCGCAGGCCTACTGA**ttctagagga

>Src Y400D

taccTCGAG**ATGCTATGCGTCAACCTCTGCAAGCCGTGTGTCCAGATCGAGAAGCCTGTAACTGAGGGGCTTTCGCACCGCACTCGCGATCAGTGGGAGATCGACAGAACGTCTTTGAAATTCGTGCGCAAACTGGGCTCCGGACAGTTTGGCGATGTCTGGGAGGGATTGTGGAACAACACAACACCTGTGGCAATTAAAACTCTGAAATCTGGTACAATGGACCCCAAGGATTTCTTAGCGGAAGCCCAGATCATGAAGAAACTGCGCCACACCAAGCTTATACAGTTGTACGCTGTCTGCACTGTTGAGGAGCCTATCTATATTATCACAGAGTTAATGAAGCACGGTTCACTGTTGGAATATCTCCAAGCCATTGCAGGCAAGGGTCGTAGCCTTAAAATGCAAACTCTGATTGATATGGCAGCGCAAATAGCTGCTGGCATGGCTTACTTGGAGTCCCAGAATTATATTCATAGGGATTTAGCGGCGCGCAATGTACTGGTAGGCGATGGAAACATCGTCAAAATCGCCGACTTTGGTTTAGCTAGGCTCATCAAGGAGGACGAAGACGAGGCGCGGGTAGGCGCCAGATTTCCCATAAAATGGACCGCTCCAGAGGCTGCTAACTACAGCAAATTCTCAATAAAATCGGATGTTTGGAGCTTTGGCATTCTTCTCACAGAACTGGTCACCTACGGACGCATACCATATCCAGGCATGACCAACGCTGAGGTGCTAACGCAAGTGGAGCACGGCTATCGAATGCCGCAACCTCCCAACTGCGAGCCGCGCCTGTATGAGATTATGCTGGAATGTTGGCACAAGGACCCCATGCGCAGACCCACGTTTGAGACGCTACAATGGAAACTGGAAGACTTCTATACATCTGATCAGAGCGACTtCAAAGAGGCGCAGGCCTACTGA**ttctagagga

>SrcDead::dGBP1-HA

GAATTCAAA**ATGCTATGCGTCAACCTCTGCAAGCCGTGTGTCCAGATCGAGAAGCCTGTAACTGAGGGGCTTTCGCACCGCACTCGCGATCAGTGGGAGATCGACAGAACGTCTTTGAAATTCGTGCGCAAACTGGGCTCCGGACAGTTTGGCGATGTCTGGGAGGGATTGTGGAACAACACAACACCTGTGGCAATTATGACTCTGAAATCTGGTACAATGGACCCCAAGGATTTCTTAGCGGAAGCCCAGATCATGAAGAAACTGCGCCACACCAAGCTTATACAGTTGTACGCTGTCTGCACTGTTGAGGAGCCTATCTATATTATCACAGAGTTAATGAAGCACGGTTCACTGTTGGAATATCTCCAAGCCATTGCAGGCAAGGGTCGTAGCCTTAAAATGCAAACTCTGATTGATATGGCAGCGCAAATAGCTGCTGGCATGGCTTACTTGGAGTCCCAGAATTATATTCATAGGGATTTAGCGGCGCGCAATGTACTGGTAGGCGATGGAAACATCGTCAAAATCGCCGACTTTGGTTTAGCTAGGCTCATCAAGGAGGACGAATACGAGGCGCGGGTAGGCGCCAGATTTCCCATAAAATGGACCGCTCCAGAGGCTGCTAACTACAGCAAATTCTCAATAAAATCGGATGTTTGGAGCTTTGGCATTCTTCTCACAGAACTGGTCACCTACGGACGCATACCATATCCAGGCATGACCAACGCTGAGGTGCTAACGCAAGTGGAGCACGGCTATCGAATGCCGCAACCTCCCAACTGCGAGCCGCGCCTGTATGAGATTATGCTGGAATGTTGGCACAAGGACCCCATGCGCAGACCCACGTTTGAGACGCTACAATGGAAACTGGAAGACTTCTATACATCTGATCAGAGCGACTTCAAAGAGGCGCAGGCCTAC**CGGCCGTCGCTCGAGATGGCCGACGTGCAGCTCGTGGAATCCGGAGGAGCACTGGTCCAGCCTGGGGGATCACTGAGACTGTCTTGTGTCGCTTCTGGCTTTCCTGTGAATCGGTACTCTATGAGATGGTACAGGCAGGCACCTGGAAAAGAGCGAGAATGGGTCGCCGGAATGTCATCTGCTGGCGATAGGAGTTCCTACGTGGATAGCGTGAAAGGACGGTTCACCATTAGAAGGGACGATGCTAGAAACACCGTGTACCTCCAGATGAACTCCCTGAAACCTGAGGACACTGCTGTGTACTACTACAACGTCAATGTGGGCTTTGAATACTGGGGACATGGCACACAAGTGACTGTTAGTTTTTACCCCTACGACGTGCCCGACTACGCCTAAACCGGTAAAT

>Src Y400E::dGBP1-HA

gaattcaaa**ATGCTATGCGTCAACCTCTGCAAGCCGTGTGTCCAGATCGAGAAGCCTGTAACTGAGGGGCTTTCGCACCGCACTCGCGATCAGTGGGAGATCGACAGAACGTCTTTGAAATTCGTGCGCAAACTGGGCTCCGGACAGTTTGGCGATGTCTGGGAGGGATTGTGGAACAACACAACACCTGTGGCAATTAAAACTCTGAAATCTGGTACAATGGACCCCAAGGATTTCTTAGCGGAAGCCCAGATCATGAAGAAACTGCGCCACACCAAGCTTATACAGTTGTACGCTGTCTGCACTGTTGAGGAGCCTATCTATATTATCACAGAGTTAATGAAGCACGGTTCACTGTTGGAATATCTCCAAGCCATTGCAGGCAAGGGTCGTAGCCTTAAAATGCAAACTCTGATTGATATGGCAGCGCAAATAGCTGCTGGCATGGCTTACTTGGAGTCCCAGAATTATATTCATAGGGATTTAGCGGCGCGCAATGTACTGGTAGGCGATGGAAACATCGTCAAAATCGCCGACTTTGGTTTAGCTAGGCTCATCAAGGAGGACGAAGAAGAGGCGCGGGTAGGCGCCAGATTTCCCATAAAATGGACCGCTCCAGAGGCTGCTAACTACAGCAAATTCTCAATAAAATCGGATGTTTGGAGCTTTGGCATTCTTCTCACAGAACTGGTCACCTACGGACGCATACCATATCCAGGCATGACCAACGCTGAGGTGCTAACGCAAGTGGAGCACGGCTATCGAATGCCGCAACCTCCCAACTGCGAGCCGCGCCTGTATGAGATTATGCTGGAATGTTGGCACAAGGACCCCATGCGCAGACCCACGTTTGAGACGCTACAATGGAAACTGGAAGACTTCTATACATCTGATCAGAGCGACTtCAaaGAGGCGCAGGCCTAC**cGGCCGTCGCTCGAGATGGCCGACGTGCAGCTCGTGGAATCCGGAGGAGCACTGGTCCAGCCTGGGGGATCACTGAGACTGTCTTGTGTCGCTTCTGGCTTTCCTGTGAATCGGTACTCTATGAGATGGTACAGGCAGGCACCTGGAAAAGAGCGAGAATGGGTCGCCGGAATGTCATCTGCTGGCGATAGGAGTTCCTACGTGGATAGCGTGAAAGGACGGTTCACCATTAGAAGGGACGATGCTAGAAACACCGTGTACCTCCAGATGAACTCCCTGAAACCTGAGGACACTGCTGTGTACTACTACAACGTCAATGTGGGCTTTGAATACTGGGGACATGGCACACAAGTGACTGTTAGTTTTTACCCCTACGACGTGCCCGACTACGCCtaaaccggtaaat

>Src Y400D::dGBP1-HA

gaattcaaa**ATGCTATGCGTCAACCTCTGCAAGCCGTGTGTCCAGATCGAGAAGCCTGTAACTGAGGGGCTTTCGCACCGCACTCGCGATCAGTGGGAGATCGACAGAACGTCTTTGAAATTCGTGCGCAAACTGGGCTCCGGACAGTTTGGCGATGTCTGGGAGGGATTGTGGAACAACACAACACCTGTGGCAATTAAAACTCTGAAATCTGGTACAATGGACCCCAAGGATTTCTTAGCGGAAGCCCAGATCATGAAGAAACTGCGCCACACCAAGCTTATACAGTTGTACGCTGTCTGCACTGTTGAGGAGCCTATCTATATTATCACAGAGTTAATGAAGCACGGTTCACTGTTGGAATATCTCCAAGCCATTGCAGGCAAGGGTCGTAGCCTTAAAATGCAAACTCTGATTGATATGGCAGCGCAAATAGCTGCTGGCATGGCTTACTTGGAGTCCCAGAATTATATTCATAGGGATTTAGCGGCGCGCAATGTACTGGTAGGCGATGGAAACATCGTCAAAATCGCCGACTTTGGTTTAGCTAGGCTCATCAAGGAGGACGAAGACGAGGCGCGGGTAGGCGCCAGATTTCCCATAAAATGGACCGCTCCAGAGGCTGCTAACTACAGCAAATTCTCAATAAAATCGGATGTTTGGAGCTTTGGCATTCTTCTCACAGAACTGGTCACCTACGGACGCATACCATATCCAGGCATGACCAACGCTGAGGTGCTAACGCAAGTGGAGCACGGCTATCGAATGCCGCAACCTCCCAACTGCGAGCCGCGCCTGTATGAGATTATGCTGGAATGTTGGCACAAGGACCCCATGCGCAGACCCACGTTTGAGACGCTACAATGGAAACTGGAAGACTTCTATACATCTGATCAGAGCGACTtCAaaGAGGCGCAGGCCTAC**cGGCCGTCGCTCGAGATGGCCGACGTGCAGCTCGTGGAATCCGGAGGAGCACTGGTCCAGCCTGGGGGATCACTGAGACTGTCTTGTGTCGCTTCTGGCTTTCCTGTGAATCGGTACTCTATGAGATGGTACAGGCAGGCACCTGGAAAAGAGCGAGAATGGGTCGCCGGAATGTCATCTGCTGGCGATAGGAGTTCCTACGTGGATAGCGTGAAAGGACGGTTCACCATTAGAAGGGACGATGCTAGAAACACCGTGTACCTCCAGATGAACTCCCTGAAACCTGAGGACACTGCTGTGTACTACTACAACGTCAATGTGGGCTTTGAATACTGGGGACATGGCACACAAGTGACTGTTAGTTTTTACCCCTACGACGTGCCCGACTACGCCtaaaccggtaaat

>Src::dGBP-HA

gaattcaaa**ATGCTATGCGTCAACCTCTGCAAGCCGTGTGTCCAGATCGAGAAGCCTGTAACTGAGGGGCTTTCGCACCGCACTCGCGATCAGTGGGAGATCGACAGAACGTCTTTGAAATTCGTGCGCAAACTGGGCTCCGGACAGTTTGGCGATGTCTGGGAGGGATTGTGGAACAACACAACACCTGTGGCAATTAAAACTCTGAAATCTGGTACAATGGACCCCAAGGATTTCTTAGCGGAAGCCCAGATCATGAAGAAACTGCGCCACACCAAGCTTATACAGTTGTACGCTGTCTGCACTGTTGAGGAGCCTATCTATATTATCACAGAGTTAATGAAGCACGGTTCACTGTTGGAATATCTCCAAGCCATTGCAGGCAAGGGTCGTAGCCTTAAAATGCAAACTCTGATTGATATGGCAGCGCAAATAGCTGCTGGCATGGCTTACTTGGAGTCCCAGAATTATATTCATAGGGATTTAGCGGCGCGCAATGTACTGGTAGGCGATGGAAACATCGTCAAAATCGCCGACTTTGGTTTAGCTAGGCTCATCAAGGAGGACGAATACGAGGCGCGGGTAGGCGCCAGATTTCCCATAAAATGGACCGCTCCAGAGGCTGCTAACTACAGCAAATTCTCAATAAAATCGGATGTTTGGAGCTTTGGCATTCTTCTCACAGAACTGGTCACCTACGGACGCATACCATATCCAGGCATGACCAACGCTGAGGTGCTAACGCAAGTGGAGCACGGCTATCGAATGCCGCAACCTCCCAACTGCGAGCCGCGCCTGTATGAGATTATGCTGGAATGTTGGCACAAGGACCCCATGCGCAGACCCACGTTTGAGACGCTACAATGGAAACTGGAAGACTTCTATACATCTGATCAGAGCGACTACAaaGAGGCGCAGGCCTAC**cGGCCGTCGCTCGAGATGGCCGACGTGCAGCTCGTGGAATCCGGAGGAGCACTGGTCCAGCCTGGGGGATCACTGAGACTGTCTTGTGTCGCTTCTGGCTTTCCTGTGAATCGGTACTCTATGAGATGGTACAGGCAGGCACCTGGAAAAGAGCGAGAATGGGTCGCCGGAATGTCATCTGCTGGCGATAGGAGTTCCTACGTGGATAGCGTGAAAGGACGGTTCACCATTAGAAGGGACGATGCTAGAAACACCGTGTACCTCCAGATGAACTCCCTGAAACCTGAGGACACTGCTGTGTACTACTACAACGTCAATGTGGGCTTTGAATACTGGGGACATGGCACACAAGTGACTGTTAGTTTTTACCCCTACGACGTGCCCGACTACGCCtaaaccggtaaat
